# Evaluation of an internet of things device for isothermal molecular detection

**DOI:** 10.1007/s15010-025-02581-1

**Published:** 2025-06-13

**Authors:** Manfred Weidmann, Jesus Bueno Alvarez, Steffen Zinn, Ruslan Ibragimov, Mohammed Hasani, Elena Graf, Aiko Weber, Frida Arrey, Marcelina Marta Szczygiel, Zuzanna Kowalewska, Rea Maja Kobialka, Arianna Ceruti, Ahmed Abd El Wahed, Michael Diebold, Christian Wahnes

**Affiliations:** 1 Midge Medical GmbH, Berlin, Germany; 2 Institute of Animal Hygiene and Veterinary Public Health, Leipzig University, An den Tierkliniken 43, D-04103 Leipzig, Germany

**Keywords:** SARS-CoV-2, Point of care test, Home test, Recombinase polymerase amplification, Internet of things

## Abstract

We describe a low-cost up scalable molecular test platform providing accessibility to frequent testing - the prerequisite to control infectious epidemics. The device uses the Multi-Spectral Digital Sensor AS7341 in a small fluorescence spectrometer which communicates with a Smartphone App via Bluetooth, which passes on the raw data to outsourced computation in the cloud via the internet. This very stable setup allows easy to use on site testing of individual samples and anonymised data analysis while providing the basis for direct result transmission of a rapid isothermal molecular diagnostic test result not only to the user but also to public health bodies. This internet of things (IoT) platform allows to collect real molecular test data from users with usability and industrial up scalability at the heart of the design. The device characteristics are described in detail and the performance for a rapid SARS-CoV-2 Recombinase Polymerase Amplification assay achieved 98.6% sensitivity and 98% specificity testing 148 positive and 501 negative samples.

**Supplementary Information:**

The online version contains supplementary material available at 10.1007/s15010-025-02581-1.

## Introduction

In the face of infectious diseases in general and particularly emerging infections and neglected tropical diseases diagnostics are central and fundamental to quality health care, however up 47% of the global population has little to no access to diagnostics. Point-of-care and/or patient self-testing, in combination with patient self-sampling has the potential to solve the last mile problem in providing access to diagnostics close to the patient in poor, rural, and marginalized communities where hitherto diagnostics are absent [1].

Suggested definitions for Point-of care tests (POCT) are “a medical test conducted at or near the site of patient care” or as “all tests performed outside the walls of the central laboratory” [2]. This includes use concepts for community outreach settings, primary health facilities including a GP`s practice, or district or satellite laboratories delivering specialised services (e.g. weekend emergency diagnostics for acute and possibly life-threatening infections). In resource limiting settings however POCT very often are not an extension of existing medical care but essentially provide access to healthcare provision were it otherwise dosen`t exist.

The Covid-19 pandemic has led to a significant increase in the awareness of the necessity and efficiency of diagnostic testing outside of centralised laboratories for contact tracing or general epidemic control [3]. Intensive research and development of point-of-care systems has been going on in academia and industry, yet the existing systems are far from delivering on the insights gained during the Covid-19 pandemic.

A recent review on point of care (POC) systems can only list devices with a price tag of 2500–8000$ which would allow high throughput testing in decentralised health care locations while a home PCR test with internet connectivity under development by Co-DX is discussed with a predicted retail price of below 1000$ [2]. Lab-on-a-chip developments although impressive very often still amount to chip-in-the-lab concepts with ancillary equipment requiring the attention of specially trained staff. The major disadvantage of most concepts however is that they have not been designed with the end-user needs in mind and lack an appreciation of the needs for integration, standardization, economy of scale for mass production and market and the added value the introduction of the system would bring to the diagnostic need [4]. The Internet of Things (IoT) describes a network of physical items linked to each other by means of the internet integrating information and real world objects to transmit information to users [5]. A recent review states that in medical research IoT is still in its infancy has not yet left the experimental stage and more than half have of developed applications have not yet been evaluated [6].

Novel CRISPR-Cas enzyme enhanced molecular isothermal cascade amplification systems generated great interest [7, 8]. Some use downstream transcription and CRISPR-Cas supported transactivation (i.e. digestion) of signal generating reporter molecules (CREST (PCR/Cas13) [9, 10]; SHERLOCK (RPA/Cas13 or Cas12) [11–13]). Other systems use CRISPR guide RNAs as direct probes to detect amplificates, which also induce transactivation of reporter molecules such as DETECTR in LAMP(Cas12) [14], and RPA (Cas12a) [15, 16], and HOLMES in PCR [17]. The latter two examples have been adapted to one-tube systems including. A major drawback however appears to be that the limit of CRISPR to cleave probes is at the picomolar level, which is not enough for highly sensitive detection [8]. Sherlock Biosciences is now pursuing the use of a different sensing system altogether called INSPECTER [18].

A recent analysis of all isothermal methods described since 1990 concluded that Recombinase Polymearse Amplifictaion (RPA) as a most desirable and promising technique for POC applications due amongst other features its specificity achieved by short amplicon size, the use of a specific hybridisation probe, and a short amplification time [19]. We adapted a a robust SARS-CoV-2 RT-RPA assay to our platform.

Here we describe a very small footprint diagnostic test station (9.2 × 6.7 × 5.0 cm) that facilitates the detection of pathogen detection in a simple and robust manner. It is built from off the shelve parts of the mobile phone industry to meet the requirements mentioned above by mature yet cost effective technologies. Design facilitates standard industry processes and such can easily be upscaled to match the need for a larger fleet of near patient testing devices at moderate costs.

**Fig. 1:**
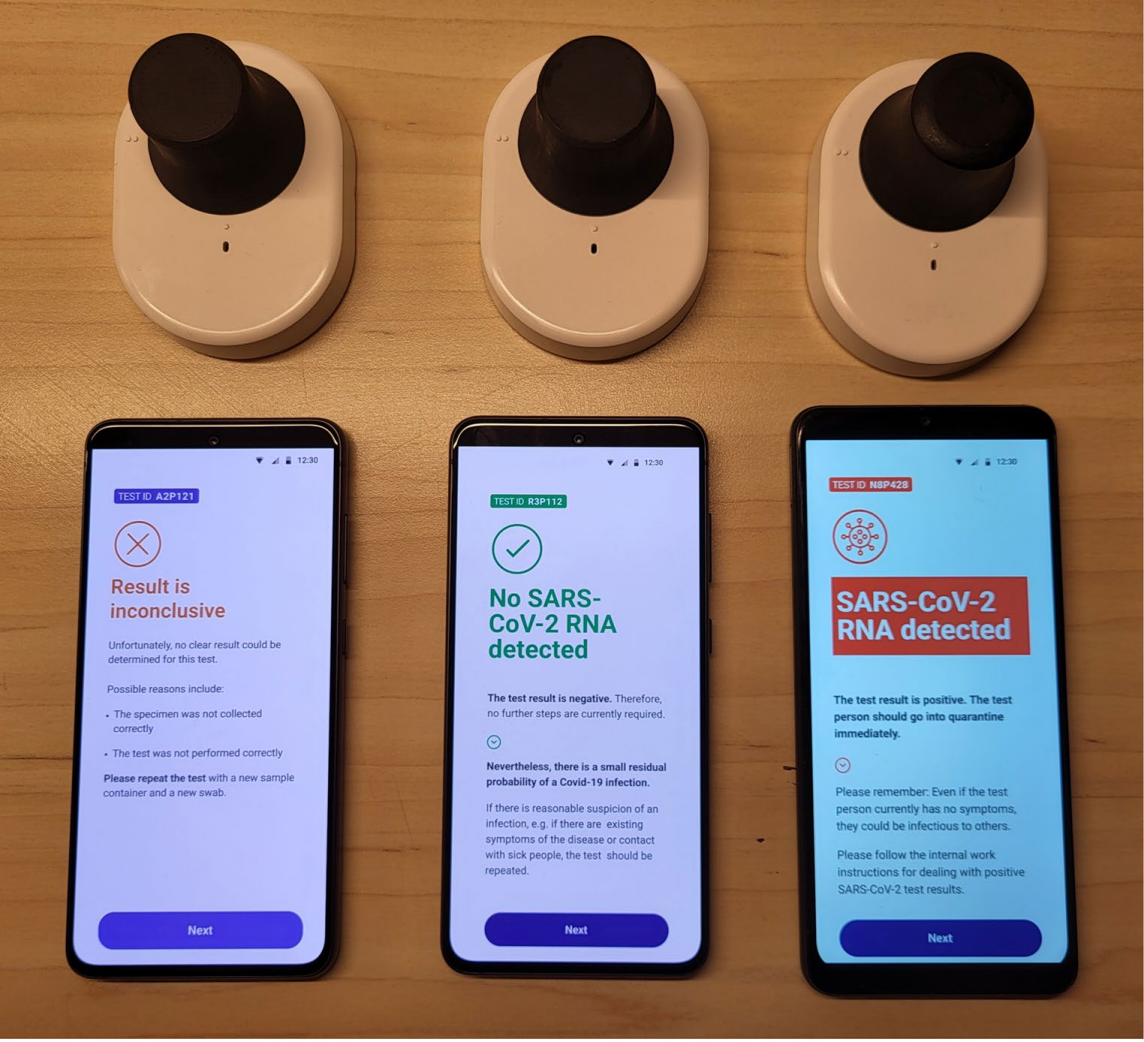
Test station. Test station with three result options displayed in the smartphone application

The battery USB chargeable device allows for 6 independent test runs when fully charged. An optical unit measures fluorescence of isothermal amplification reactions in standard 200 µL PCR tubes using a Light Emitting Diode (LED) and an onboard and the onboard multi-spectral digital sensor AS7341. An independent onboard firmware wirelessly (Bluetooth) addressed via a smartphone application sends raw data to the application which communicates these to a backend server (Wi-Fi) for analysis. The smartphone application receives the results as either positive, negative or inconclusive immediately after analysis (Fig. 1).

The device was developed in a typical stage gate process according to EN ISO13485. The platform sections software, hardware and biochemical assay went through verification and validation and the data were summarized and CE-IVDD certification was achieved in May 2022. In the following we describe key experiments characterizing the fluorescent readout and signal correction, temperature control and present data on the overall performance of the platform using a SARS-CoV-2 RT-RPA with clinical samples.”

## Materials and methods

### Fluorescence read out of test station

To characterize the Multi-Spectral Digital Sensor AS7341 (AMS, Premstätten, Austria), fluorescence dye BAM2 (Bundesanstalt für Materialforschung und Prüfung, Berlin, Germany) and FITC (Fluorescein NIST-Traceable Standard, Thermofischer Scientific, Heningsdorf, Germany) were diluted in HEPES buffer in a 10-fold dilution series. Two µl were measured in fluorospectrometer Nanodrop 3300 (Thermo Scientific, Langenselbold, Germany) and 50 µl were measured on the AxxinT8 (Axxin, Melbourne, Australia) and test stations (*n* = 3).

**Fig. 2:**
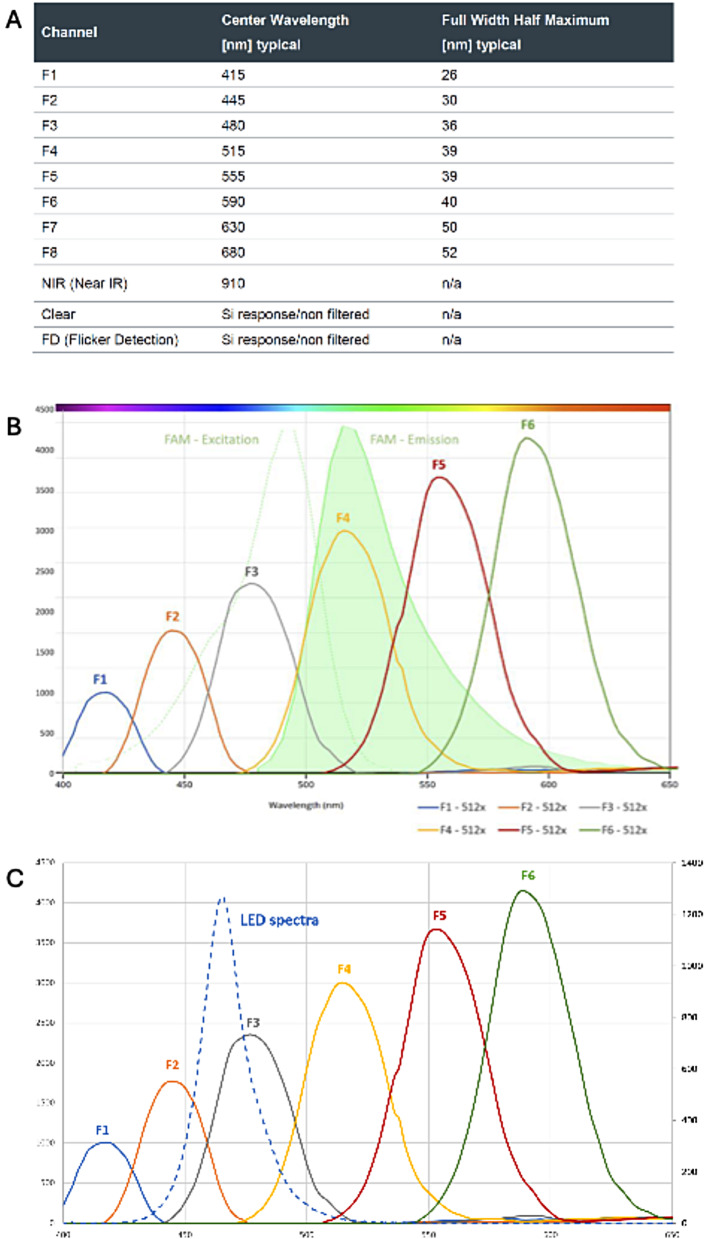
Eleven Channel Multi-Spectral Digital Sensor (AS7341). **A**: Overview channels; **B**: Fluorophore emission overlap; C: Responsivity & LED emission of test station

Channels F1 and F2 of the AS7341 sensor do not overlap with the emission spectrum of the F4 channel used to measure fluorescent RPA reaction signals (FAM = F4_RPA) and were considered as candidates to approximate the non-fluorescent background of channel F4 derived from LED light reflection and scattered light (F4_SCT) (Fig. 2A-C). The following assumptions were made:

(i) The primary F4 signal can be expressed as *F4 (t) = F4_RPA (t) + F4_SCT (t)*, where *t* denotes time during a run (Eq. 1). *F4_RPA* denotes here the fluorescence signal produced during an RPA reaction.
(ii) F4_SCT does not remain constant during the reaction, F4_SCT(t) can be dynamically approximated from channels that do not contain RPA-derived signal, such as F1. This requires that: Channel F1 (and F2) do not capture fluorescence signal: *F1 (t) = F1_SCT (t.* (EQ.2).
(iii) There is a linear relationship between non-RPA signal of different channels. F4_SCT/min{F4_SCT} = k * F2_SCT/min{F2_SCT}, where *k ~ 1* is a constant (Eq. 3).
(iv) Due to the overlap of the LED emission with the F4 channel (Fig. 2C) the non-RPA component of F4 is always larger than 0: *F4_SCT(t) > 0 t* (Eq. 5).

To show (ii) evolution of F_SCT and (iii) linear relationship between non-RPA signals, negative runs were analysed and raw data and normalised data plotted. To show that (iv) F4_SCT is always larger than 0, additionally dark System Suitability Test (SST) series with no tube (and sample) and only a cap on top of the optical block were run and analysed. The following parameters for gain, integration time and LED intensity were used (AGAIN = 512x; ATIME = 4; ASTEP = 65534, LED intensity = 100%).

### Software infrastructure and algorithm

**Fig. 3:**
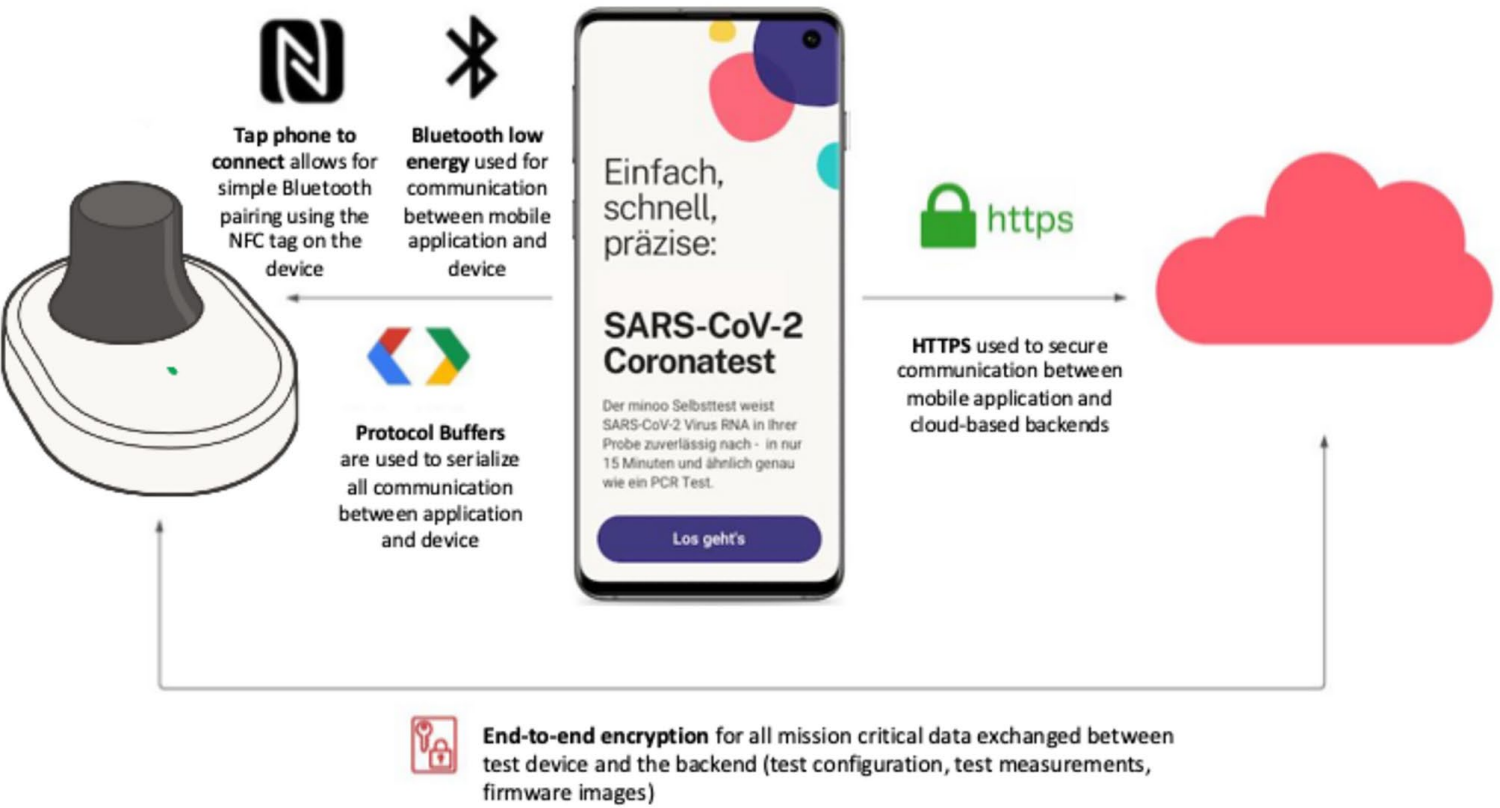
Software infrastructure. Firmware on the test station, Android App on Smartphone, backend with analysis algorithm. An anonymous code is generated for each test when scanning the test pouch QR code

The test station executes the test procedure and provides data through a firmware embedded in the device. An Android mobile application is the interface to the user, allowing for starting and controlling a test run. It provides instructions to the user, initiates the test, and facilitates communication between the device and the cloud infrastructure (backend). The backend stores and manages test and user data, calculates test results, and ensures compatibility of components, serves as a single source of truth for all data (Fig. 3). Data transfer is encrypted and authenticated, securing the transmission against tampering.

### Reaction temperature control

To test the stability and robustness of the of the simple heating coil and temperature sensor system experiments were performed immediately outside the operational limit range (21–28 °C and 20 − 70% RH (non-condensing), (Table S1), defined for the CE product, at 20 °C, 23 °C and 30 °C. The tests were conducted in a temperature-controlled room equipped with air conditioning, and ambient temperature was monitored using a calibrated Testo 174 H data logger (measurement accuracy ± 0.5 °C). For temperature calibration of the devices during manufacturing, sample temperature was monitored using PT1000 Class A sensors, placed in the sample tube. The analysis was performed by plotting the temperature readouts of reaction temperatures typically used in RPA reactions (42 °C). *n* = 8 devices were used for each reaction temperature.

### Device robustness

To evaluate the operational life of the test station, a series of 1000 consecutive test runs was performed on a group of nine devices. Following these cycles, the performance of the devices was assessed using lyophilized proprietary RT-RPA beads and 10^5^ in vitro RNA molecules (see below), corresponding to the system’s targeted limit of detection. Testing was conducted within the system’s defined operational temperature and humidity limits (Table S1).

### RNA amplification

For real time RT-PCR (rtRT-PCR) the TATAA GrandPerformance SARS-CoV-2 detection kit (TATAA Biocenter AB, Göteborg, Sweden) containing published SARS-CoV-2 primers targeting the RdRP gene [20] was used on the Qiagen Rotor-Gene cycler (Qiagen, Hilden Germany). For Recombinase Polymerase amplification a RT-RPA biochemistry as described in patents and publications by TWIST DX was developed and produced as a lyophilized RPA pellet. It contained previously described RPA primers targeting the RdRP. An in vitro SARS-CoV-2 RdRP gene RNA standard (ivRNA) confirmed by rtRT-PCR was used (GeneExpress, Berlin, Germany) [21]. A positive control pellet containing 10^5^ RNA molecules of this ivRNA was also developed. Accelerated and real time studies showed storage stability of these lyophilized RPA pellets and positive control pellets at room temperature for 8 months and initial signs of degradation 36 weeks if stored at 32°C. Both pellets were provided in a kit (Figure S1).”

### Generic extraction and amplification protocol

Extraction of SARS-CoV-2 RNA was performed in the rehydration buffer with added detergent. A throat swab dipped into 600 µl rehydration lysis buffer (RHL) in the lysis tube and twirled for 10 s washed out the sample into the RHL. The RHL buffer consists of the original RPA recipe including 14mM Mg-acetate, supplemented with 0.45% of detergent NP40-substitute CAS 9016-45-9 (Sigma, Germany). 50 µl of the RHL were then transferred into the reaction tube containing the RPA pellet with a 100 µl pipette and solution of the pellet was supported by gently aspirating the suspension up and down 5x (Figure S2).

### Analytical sensitivity and specificity testing

The ivRNA standard was used to determine the sensitivity of the RT-RPA assay. It showed concordance in an overlapping range of 10^4^-10^3^ molecules / reaction with the First WHO International Standard for SARS-CoV-2 RNA 20/146 (National Institute for Biological Standards, Potters Bar, United Kingdom) and the ivRNA was used to determine the limit of detection of the test (*n* = 5/ concentration point). An extensive specificity assessment of the RdRp RT-RPA assay used has already been reported [21].To confirm the analytic specificity, cell culture RNA of seven different strains of coronavirus (European Virus Archive global panel Ref-SKU: 011 N-03868) i.e. *Betacoronavirus pandemicum* (SARS-CoV, SARS-CoV-2, SARS-CoV-2 Omicron), *Betacoronavirus cameli* (MERS-CoV), *Alphacoronavirus amsterdamense* (HCoV NE63), *Alphacoronavirus chicagoense* (HCoV 229E), and *Betacoronavirus gravedinis* (HCoV OC43) [22]), were tested in triplicate.

### Diagnostic sensitivity and specificity testing

One hundred SARS-CoV-2 positive and 501 negative clinical samples in an inactivating GITC containing buffer were provided by a diagnostic laboratory providing SARS-CoV-2 PCR testing for hospitals (Labor Dr. Fenner & Kollegen MVZ, Hamburg, Germany) from which routinely RNA would have been extracted by a silica column based kit. To adapt this to our RHL buffer system, we remove GITC by adding 120 µl of the samples to a reverse chromatography column of a EchoCLEAN kit (BioEcho Life Sciences, Cologne, Germany). Five microlitre of the eluate were used in a 20 µl PCR volume. To mimic swab use in the RHL, 110 µl of the eluate was spiked into the RHL buffer by mixing t with 190 µl of water and 300 µl of RHL 2x buffer. Fifty microllitre of this mix were added to the RPA pellet. Overall, the sample dilution was 1: 4 and 1:5.5 in rtPCR and RPA respectively. Ninety-two samples scored positive by rtRT-PCR (CT 22.7–29.5). To generate a higher CT spread > CT 30, seven positive samples picked at random were additionally diluted in a 2-fold dilution series from from 1:10 − 1: 640. To preserve the sample matrix background eluates of negative samples were used as the diluent. This generated an additional 56 samples (CT 32.98–38.83) in a Gaussian distribution (Fig. S3). All 148 rtRT-PCR positive samples (CT 22.7-38.83) were used to determine diagnostic sensitivity and specificity.

### Algorithm and statistical analysis

Test station raw data were processed using the proprietary Kassandra algorithm to classify results and to determined TT values. Kassandra is a proprietary Python-based algorithm which performs three main steps: data processing, i.e. removal of background from the primary channel and quality checks; feature extraction and classification: positive, negative, or inconclusive which are reported to the smartphone application.

rtRT-PCR raw data were analysed with the Qiagen Rotor-Gene Q Software 2.3.1.49. Data were analysed in EXCEL, PRISM, and an online calculator: https://www.scistat.com/statisticaltests/ diagnostic_test.php to determine diagnostic sensitivity, specificity, positive predictive value (PPV), and negative predictive value (NPV). RStudio version 1.3.1093 (RStudio, Boston, MA, United States), was used to perform probit regression and calculation of the LOD. To visualize the differences in the data between the test results principal component analysis (PCA) was applied to the primary algorithm features used for classification using Python with the packages pandas and scikit-learn.

## Results

### Device characteristics

To characterize the fluorescence read out of the 11-Channel Multi-Spectral Digital Sensor AS7341 in the test device standard dye BAM2 and FITC dilutions were comparatively analysed on the Nanodrop, Axxin and test devices. The emission peaks of BAM2 (512 nm) and FITC (512 nm) overlapped with the channel F4 of the spectrometer. Usual RPA reactions lead to fluorescence levels in the fluorospectrometer (gain 5) in the range 500 to 1500 RFU which corresponded to BAM2 concentrations at 50, 100 and 150nM subsequently used to select the ideal combination of signal parameters. Results indicated that the signal to water (S/W) ratio stayed the same, independently of parameter set and gain. The best signal to noise ratios (SNR) were achieved at an LED power of 100%.

It is generally accepted to define the LOD as the concentration at which the SNR is equal to 3 (for qualitative purposes) or equal to 10 (for quantitative measurements). In our case, the SNRs obtained for all dilutions were over these defined thresholds. S/W results show that for concentrations lower than 10nM the sensitivity is reduced. In other words, reliable quantitative measurements can be performed for concentrations higher than 10nM. Saturation was reached for concentrations of approximately 5000nM.

Spectrometer readouts from the F4 channel do not only include fluorescence signal resulting from the amplification itself but also ‘background light’ derived from LED light reflection, scattered light, etc., which could mask the RPA signal, and thus, lead to low sensitivity in the system.

This background signal is dependent on the setup and is affected by changes in the sample that occur along a test run such as, bubble formation, condensation, and changes in chemical structures e.g. solving of the polymer in the RPA reaction. Since scattered light is dependent on the wavelength, channels F1 and F2 closer to F4 were considered appropriate to mathematically derive true F4 signal.

### Correction of primary fluorescence signal

**Fig. 4:**
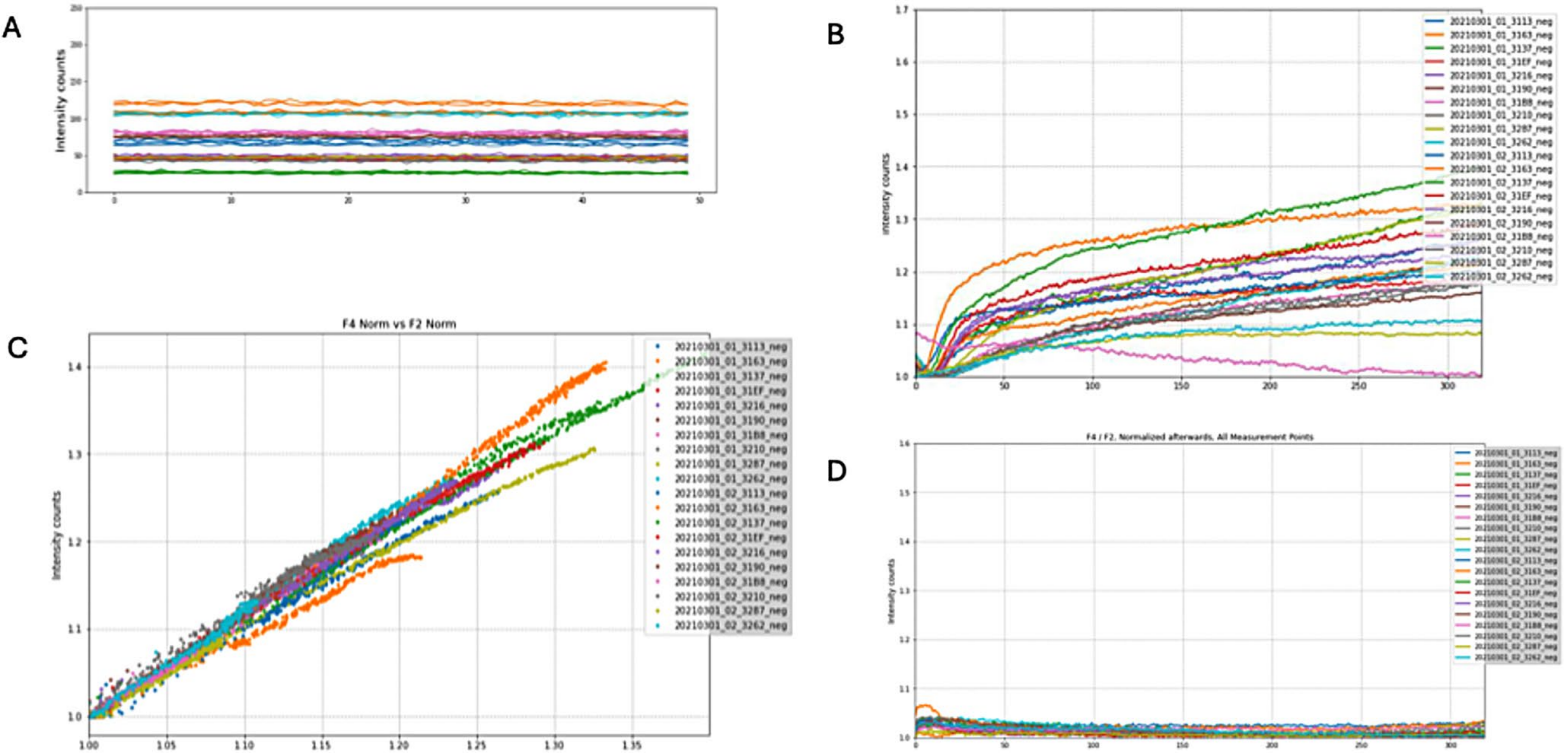
Correction of primary fluorescence signal. A: SST experiments showing constant background signal (*n* = 20). Read out of negative runs. B: F4 signal of negative runs changing over time and normalized by the minimum (reached after 150 s) (*n* = 20). C: Signal of spectrometer channel F4 vs. F2 with a slope of approx. -1 (*n* = 20). D: Negative runs normalized using Eq. 4 results in flat curves

The SST experiments confirmed that there is always a scatter light signal > 0 in channel F4 (Fig. 4A) and additionally the scatter light signal is not constant, and changes along a negative test run (Fig. 4B). The scattered component in channels follows the linear relationship expressed in Eq. 3. and *k* was approximated as 1 (Fig. 4C). The same was observed in the initial negative phase of the positive reactions. Thus, for a correct approximation of F4_SCT this approximation the following equation was derived (F4/F2)’ = 1 + F4_RPA (t) / F4_SCT (t) (Eq. 4). The implementation of this normalisation term indeed resulted in flat negative curves (Fig. 4D) and positive curves with an initial negative stretch before the true fluorescent signal rises (not shown).

**Fig. 5:**
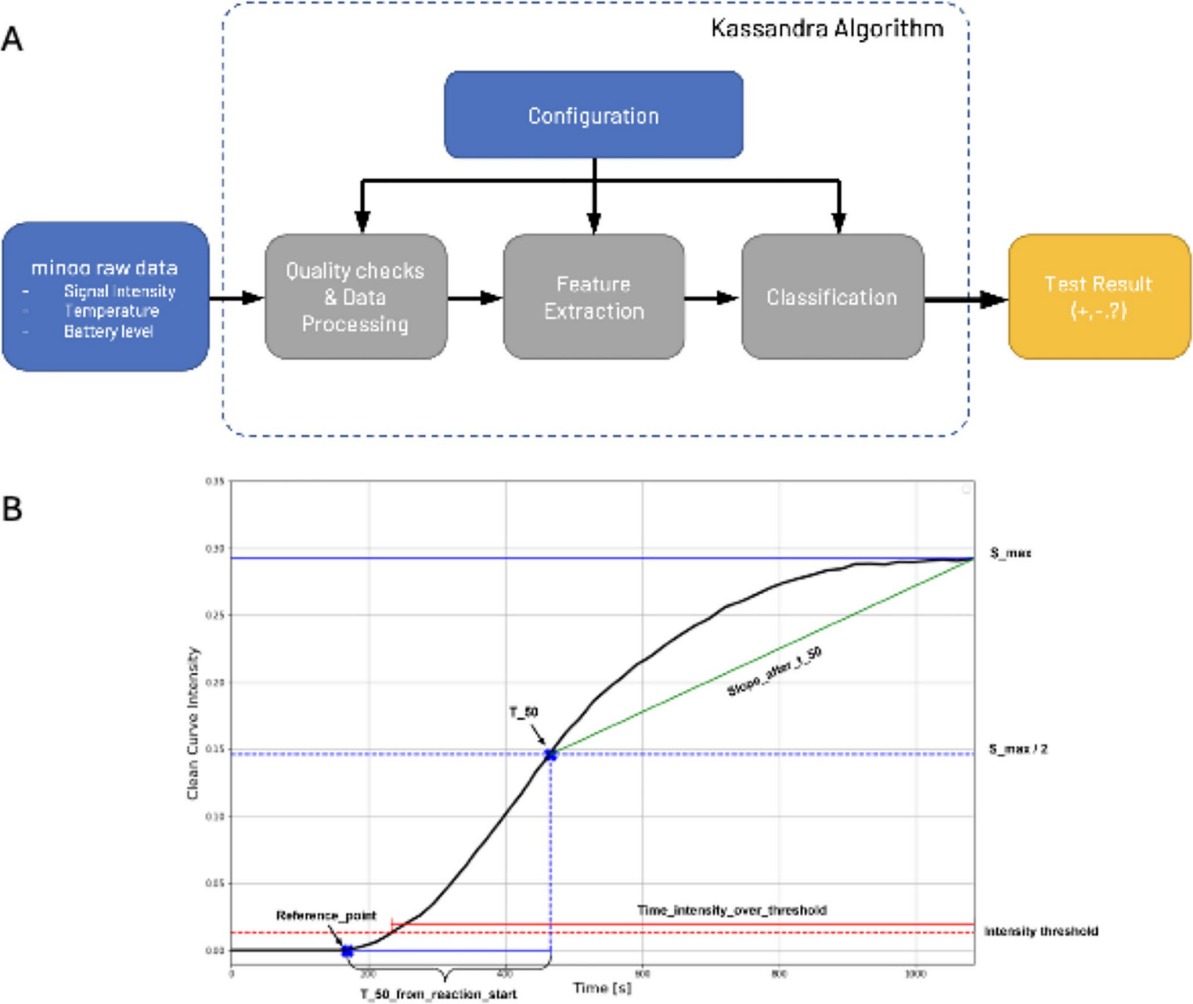
Kassandra algorithm overview

This normalisation was included into a bespoke algorithm designed for the comprehensive analysis and processing of data from the test station termed Kassandra. It classifies the test runs into either positive, negative or inconclusive. After rigorous quality checks, the algorithm extracts key feature values and uses empirical defined thresholds and weights to calculate a test result. It additionally calculates a TT value to allow comparison to TT values of other devices (Fig. 5). Validated with a SARS-CoV-2 dataset from the clinical study described below, Kassandra demonstrated a remarkable accuracy rate of 97.8%.

### Temperature control

**Fig. 6:**
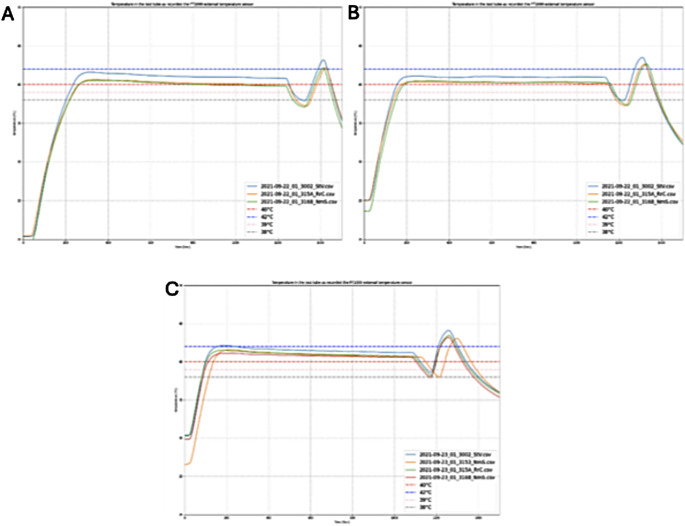
Temperature in the reaction chamber at various ambient temperatures. **A**: 20 °C, **B**: 23 °C, C: 30 °C

We conducted a series of experiments to assess the variability of the sample temperature in response to changes in ambient temperature. Our results showed a variation of + 0.58 °C (SD ± 0.06 °C) in sample temperature when the ambient temperature increased from 20 °C to 30 °C (Fig. 6). These findings highlight the robustness of the device’s simple temperature control setup, particularly at elevated room temperatures. To ensure accurate sample temperature regulation, each device is individually calibrated during manufacturing to achieve 42 °C within the sample container.

For the robustness tests three sets of runs were conducted at temperatures exceeding 30 °C with humidity levels above 60%, while another three sets of runs were carried out at 20 °C with a humidity level of 33%. In all cases the results indicated that all devices successfully produced the expected outcomes after completing the 1000-run test.

### Analytical sensitivity and specificity of the SARS-CoV-2 RPA assay

The tested virus panel yielded positive reactions exclusively for SARS-CoV-2 and SARS-CoV-2 Omicron. The SARS-CoV-2 RT-RPA pellet was tested on the tested device using a molecular standard. An initial 10-fold dilution 10^6^ -10 RNA molecules/ reaction (*n* = 5) scored 5/5 above 10^4^. A 5-fold dilution range starting from 10^5^ RNA molecules/ reaction (*n* = 24) scored 24, 20, 7, 3, and 3 positive out of 24 for 10^5^, 2 × 10^4^, 4 × 10^3^, 8 × 10^2^, and 1.6 × 10^2^ RNA molecules/ reaction respectively. A probit analysis using this data set calculated a limit of detection of 26,101 RNA molecules per reaction (Probit plot not shown, results plotted by poly component analysis in Fig. S2).

### Diagnostic sensitivity and specificity

**Fig. 7:**
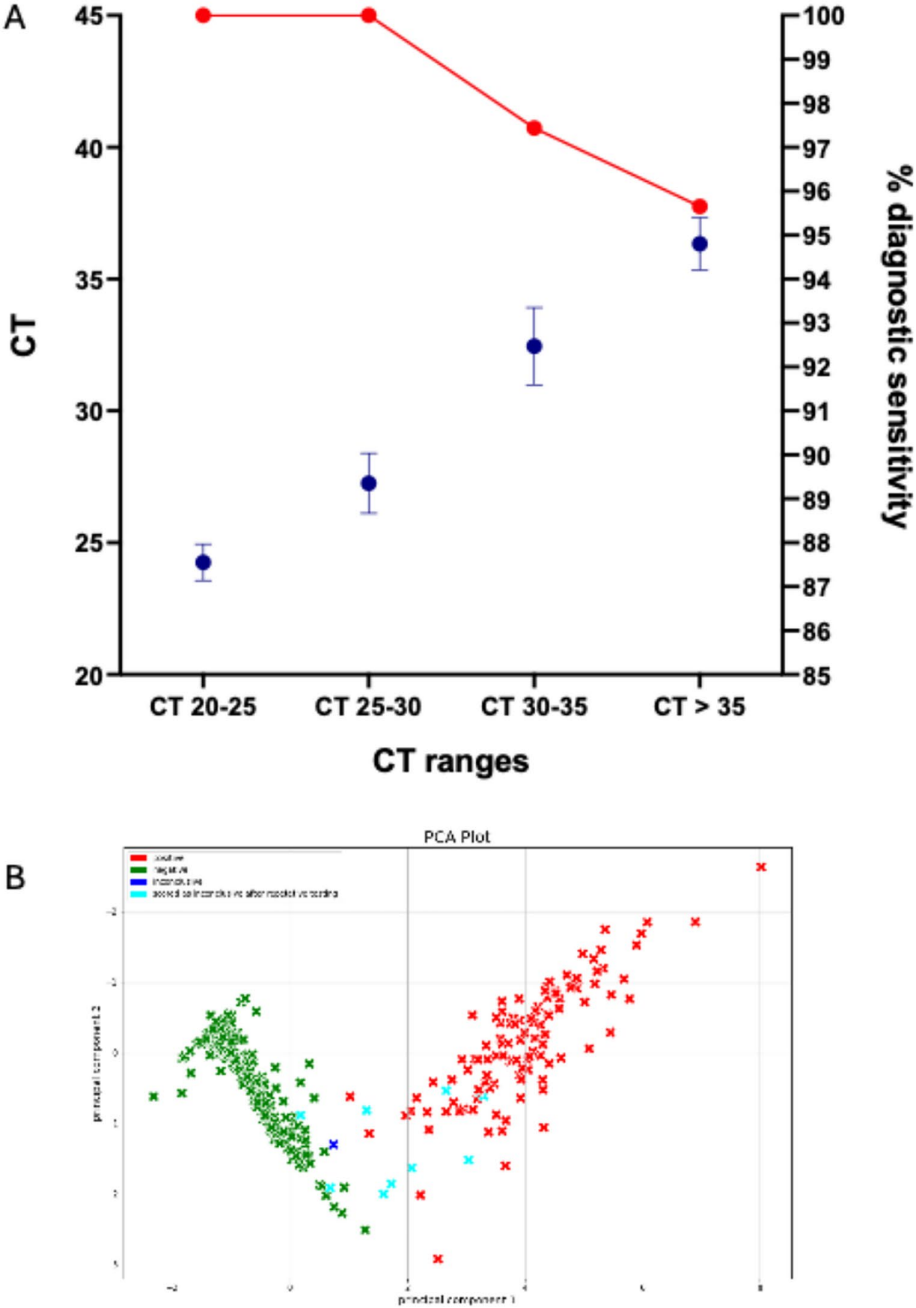
A: Diagnostic sensitivity. CT range in CT groups plotted on left y-axis (*n* = 12 / 74 / 9 / 23) versus sensitivity in groups plotted on right y-axis (1.0, / 1.0 / 0.974 / 0,957). PCR positive samples < CT: 86 (range 22.7–29.6), samples > CT 62 (range 30.1–38.8); B: Partial component analysis of all diagnostic sensitivity results. Green: negative, light blue: inconclusive, red positive

In order to assess the diagnostic sensitivity and specificity of the SARS-CoV-2 RT-RPA of the test platform, and following the relevant MDCG guideline [23] a total of 649 clinical samples were tested (148 positive and 501 negative clinical samples). To mimic swab use inactivating agent GITC was removed from clinical samples, which were then spiked into 2x RHL buffer and subsequently tested for SARS-CoV-2. The sample with the highest reference CT detected was CT 38.83. The highest CT 38.83 detected in a clinical sample by the test platform represents an LOD of 1.59 × 10^5^ RNA molecules / ml as quantified by real time PCR and corresponds to the value of 5.01 × 10^5^ RNA molecules / ml determined for the analytical sensitivity by probit analysis. The analysis of the results yielded a diagnostic sensitivity of 98.6%, a diagnostic specificity of 98.0%, and an accuracy of 98.1% Table 1. Figure 7)

**Table 1:** Diagnostic sensitivity and specificity

Analysis values	PPV	NPV	sensitivity	specificity	test platform RPA	Reference PCR	
estimate: 95%CI:	0.936 [0.88;0.96]	0.996 [0.98;0.99]	0.986 [0.95;0.99]	0.98 [0.96;0.99]		**pos**	**neg**
					**pos**	146	10
					**neg**	2	491

## Discussion

Access to healthcare services at home (e-Health) through cost effective IOT applications could empower patients to contribute to health monitoring and essentially health promotion and enhanced wellbeing. This motto of IOT concepts in medicine imagines a huge number of sensors transmitting data from many locations analysed rapidly to provide services [6].

Our robust spectrometer is a digitally connected readout device for fluorescence signals generated by an isothermal RPA operating at merely 42 °C while analysis of the data is outsourced to a backend (cloud). Since initial experiments indicated an effect of scattering light on the fluorescence signal the correction we chose allowed to reduce that effect to a minimum as evidenced by the flat negative control lines in Fig. 4D.

The device was shown to robustly perform its core functions of heating even in temperature conditions outside its specified limits above 28 °C (Fig. 6). Overall testing of the device demonstrated the detection and analysis of a specific fluorescence signal generated by the RPA reaction at a diagnostic sensitivity of 5.01 × 10^5^ RNA molecules / ml for SARS-CoV-2 detection from throat swabs (Table 1; Fig. 7). This sensitivity ranges in the order of magnitude of sensitivity of antigen detection tests [24]. A limitation of our study is that we did not test samples of patients with other respiratory diseases.

Early studies in the Covid-19 pandemic on SARS-CoV-2 detection in patient samples (reviewed in [25]) concluded that in upper respiratory tract samples viral load peaks around the onset of disease followed by a decline of two weeks until not detectable anymore. False negative results of possibly asymptomatic persons have the potential to sustain the transmission of the virus to others and viral loads were shown to be similar in asymptomatic and symptomatic individuals with faster viral clearance among asymptomatic individuals than those who are symptomatic [26; 27]. It was shown that the phase between exposure and onset of disease produces the highest false negative rates for qPCR to a minimum of 21% on day 3 post onset after which it begins to continuously rise until day 21 post onset of symptoms leaving a window of 3–5 days after onset of disease as ideal for testing with the lowest false negative rate [28]. In principle false negative rates for SARS-CoV-2 tests were due to variability in viral shedding [29] and sample type [30]. Additional analysis also showed that qPCR results with a CT > 30 did not corelate with infectious viruses in the sample [31] and that for that matter antigen test results corelated much better with the presence of infectious viruses in the sample [24; 32]. Altogether this supported the antigen testing schemes used worldwide to clear people from isolation and to perform surveillance testing.

A modelling study by Larremore et al. looking at the progression of viral load in SARS-COV-2 samples and the detection of viral RNA by molecular tests with a limit of detection ranging from 10^3^/ml or 10^5^/ml convincingly demonstrated that surveillance effectiveness depends mainly on frequency of testing and speed of reporting rather than on assay sensitivity. It was concluded that accessibility to tests, frequency of testing and sample-to-answer time are more relevant to surveillance effectiveness than the limit of detection [33]. An epidemiological model analysed testing and epidemiological data from the Covid-19 outbreak in Italy and conclusively showed that an early test and isolation strategy indeed disrupts transmission chains and reduces hospital occupancy significantly [34]. In Vienna simplifying the logistics of self-sampling by gargling and shipment of samples to PCR laboratories essentially made testing and repetitive testing accessible at high frequency [35]. Inspired by this initiative a modelling study on how this setup if transferred to Germany using a cheap PCR concept for mass testing with a time to result of 24 h concluded that if 60% of the population (50 million samples a day) had been tested in the early stage of the pandemic it would have succeeded in interrupting the transmission chains [36]. In regard to the cited literature our platform therefore offers highly accessible, frequent, and specific nucleic acid amplification testing, with a short time to result which ticks all the points made by Larremore and by the cited epidemiological studies.

The low cost, diagnostic sensitivity, fast time to result of the test device described here in combination with the potential accessibility through easily up scalable cheap production therefore combines surveillance effectiveness with the economy of scale for mass production and market.

The user of the test platform is guided through the self-sampling process by several screens of the smartphone App which is in line with studies which demonstrated that there is little difference in professional sampling and lay self-sampling with the best results obtained in supervised self-sampling [37] in Berlin, or demonstrations of self-sampling to particular groups in Malawi [38]. Also, a study from the United States demonstrated that home testing positivity rates refelected official positivity rates from national results data from August 2021 to June 2022 [39]. Given these experiences self-sampling and home testing using the test platform should efficiently provide reliable results.

The stable transmission of data via Bluetooth to the Smartphone App and via the internet to the backend is based on experience gathered for the transmission infrastructure implemented for the connection of an Implantable Cardioverter-Defibrillator [40] to the internet and was shown to work even in conditions of unstable internet availability [41]. Notably this IOT set up also allows fleet management monitoring the state of each device every time it is connected. During verification it was shown that the simple device is potentially stable for > 1000 test runs and so the device monitoring feature could actually collect the data allowing for post market surveillance (requirement of the IVDR) and a customer friendly device replacement plan if thousands of devices were to be in use.

IOTs published so far in the area of infectious disease conceptualized the use of wireless body area network (WBAN) sensors collecting vital parameters of the body of patients and the public to help monitor and control either Ebola or Chikungunya disease through analysing data at scale [6; 42; 43]. In contrast the low-cost up scalable molecular test platform is an IOT concept delivering patient generated distinct molecular test results directly to the patient. The true IOT potential of the digitally connected platform however resides in transmission of results to epidemiological platforms of public health services and their processing of the data. The interfaces have been prepared but not yet implemented and would be subject to bespoke adaptation to respective GDPR regulations.

In the US Elume Covid-19 home tests (lateral flow antigen test with Smartphone App readout) was the first to be granted Emergency Use Approval by the FDA in December 2020. In 2023 the FDA approved the first at-home over-the-counter molecular test by Cue Health (LAMP assay with Smartphone App readout) for Covid-19 using a traditional premarket review rather than emergency use pathway. In May 2024 however the FDA sent out a warning letter for the product since the change management of the test had not been compliant to guidelines as EN ISO 13,485 and CFR 820, and the company subsequently filed bankruptcy [44; 45; 46]. The regulatory framework for home testing is available but it appears that after the post Covid decline in test demand the diagnostic industry is wary of home test approaches. Additionally in general it fears competition rather than realizing that tests with low complexity, but huge accessibility could actually improve the effectiveness of diagnostic turnover in centralized laboratory diagnostics by funnelling identified cases to their centres.

A major potential area or the test platform presented here would be in infrastructure poor settings as it meets many of the conclusions of the Lancet commission report on diagnostics [1] and the majority of the REASSURED criteria [47]: It provides results in real-time; uses easy sample collection for swab samples in this case; is affordable in the sense that it outcompetes POC device pricing in basic manufacturing and up scaled manufacturing if compared to the lowest purchase prices for a POC devices (1000$) discussed by Gavina [2]; is sensitive and specific as shown by the verification data; is user friendly as the consumer utility was included in the design through technical to customer requirements, accompanying risk management and user studies; is rapid as the whole procedure from self-sampling to result takes about 23 min. It is however not device free which could be compensated through decentralised provision of the device at local pharmacies for example where users would only have to buy a test pouch. Modelling trials for production of the biochemistry in Senegal and India indicated prices of < 1$/test in up scaled production (data not shown). In principle the test platform would be deliverable to the users as device production at international electronics/mobile phone manufacturers and local biochemistry production would allow to disseminate the device at great scale.

## Electronic supplementary material

Below is the link to the electronic supplementary material.

Supplementary Material 1

Supplementary Material 2: Table S1 Operating Environment, Figure S1 Kit contents, Figure S2 User self-sampling and test work flow for SARS-CoV-2 test, Fig. S3. Distribution of CTs of diluted positive samples, Fig. S4. Principal component analysis of all analytical sensitivity results.

## Data Availability

No datasets were generated or analysed during the current study.
