# Supplementary material for: Evaluation of an internet of things device for isothermal molecular detection
Source: Infection. 2025 Jun 13;53(6):2467–80. doi: 10.1007/s15010-025-02581-1 (PMC12675774; doi:10.1007/s15010-025-02581-1)
Supplement: Supplementary file 1 — Supplementary Material 1 [file 15010_2025_2581_MOESM1_ESM.docx]

**Suplement**

Figure S1. Distribution of CTs of diluted positive samples

 
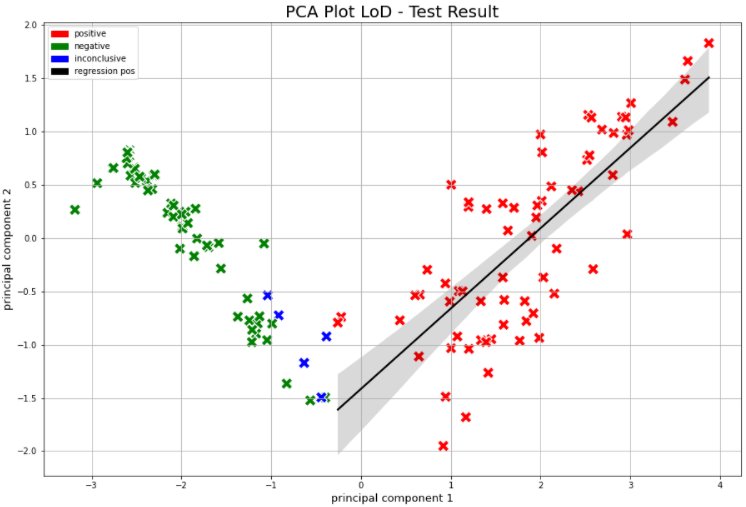


Figure S2. Principal component analysis of all analytical sensitivity results. Green: negative, Light blue: inconclusive, Red positive.
