# Supplementary material for: Evaluation of an internet of things device for isothermal molecular detection
Source: Infection. 2025 Jun 13;53(6):2467–80. doi: 10.1007/s15010-025-02581-1 (PMC12675774; doi:10.1007/s15010-025-02581-1)
Supplement: Supplementary file 2 — Supplementary Material 2: Table S1 Operating Environment, Figure S1 Kit contents, Figure S2 User self-sampling and test work flow for SARS-CoV-2 test, Fig. S3. Distribution of CTs of diluted positive samples, Fig. S4. Principal component analysis of all analytical sensitivity results. [file 15010_2025_2581_MOESM2_ESM.docx]

**Supplement**

**Table S1** Operating Environment

| Indoor/Outdoor | Indoor Use |
| --- | --- |
| Temperature | 21°C to 28°C |
| Humidity | 20% to 70% RH (non-condensing) |
| Altitude | 0 to 2000m altitude |
| Pollution degree | 2 |
| Pressure | Pressure 0.77 to 1.01 bar |
| Light Conditions | Maximum light intensity of 2,000 LUX |

**Figure S1** Kit contents: Positiv control test vial (1x), 1. Swab (10x), 2. Lysis vial (11x), 3. Test vial (10x), 4. Pipette cones (reaction tube and pipette tip are provided together in a bipartite pouch), 5. Instructions for use, P. Test station

**Figure S2** User self-sampling and test work flow for SARS-CoV-2 test

**Figure S3.** Distribution of CTs of diluted positive samples


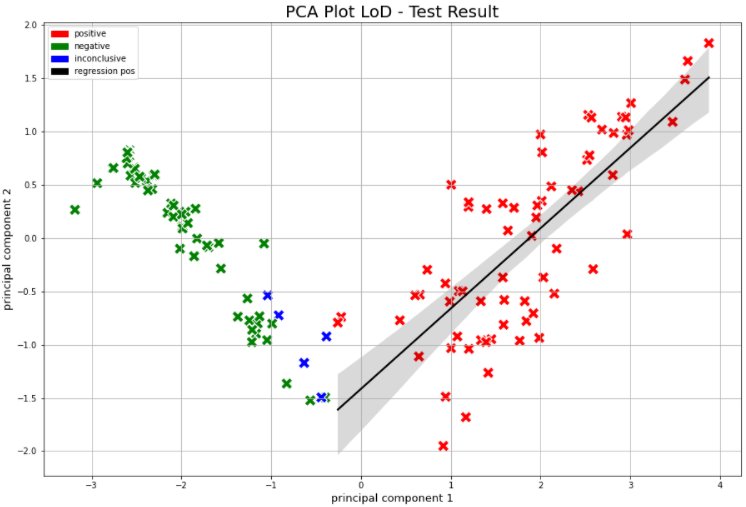


**Figure S4.** Principal component analysis of all analytical sensitivity results. Green: negative, Light blue: inconclusive, Red positive.
